# Supplementary material for: A Comprehensive Study on the Electrostatic Properties of Tubulin-Tubulin Complexes in Microtubules
Source: Cells. 2023 Jan 5;12(2):238. doi: 10.3390/cells12020238 (PMC9857020; doi:10.3390/cells12020238)
Supplement: Supplementary file 1 [file cells-12-00238-s001.zip › cells-2050741-SI.pdf]

Electrostatic interactions play essential roles in tubulin-tubulin polymerization of microtubules

*Wenhan Guo[1], Tolulope Ale[1], Shengjie Sun[1], Jason E Sanchez[1], Lin Li[1,2]**

[1] Computational Science Program, University of Texas at El Paso, El Paso, TX
[2] Department of Physics, University of Texas at El Paso, El Paso, TX

* **Correspondence:** Email: lli5@utep.edu.

Movies:

Movie1: Simulation of α/α tubulin complex (10ns)

Movie2: Simulation of β/β tubulin complex (10ns)

Movie3: Simulation of α/β tubulin complex (10ns)

Movie4: Simulation of β/α tubulin complex (10ns)
